# The Treatment of Cognitive, Behavioural and Motor Impairments from Brain Injury and Neurodegenerative Diseases through Cannabinoid System Modulation—Evidence from In Vivo Studies

**DOI:** 10.3390/jcm9082395

**Published:** 2020-07-27

**Authors:** Daniela Calina, Ana Maria Buga, Mihaela Mitroi, Aleksandra Buha, Constantin Caruntu, Cristian Scheau, Abdelhakim Bouyahya, Nasreddine El Omari, Naoual El Menyiy, Anca Oana Docea

**Affiliations:** 1 Clinical Pharmacy Department, University of Medicine and Pharmacy of Craiova, 200349 Craiova, Romania; 2 Biochemistry Department, University of Medicine and Pharmacy of Craiova, 200349 Craiova, Romania; anne_mary_07@yahoo.com; 3 ENT Department, University of Medicine and Pharmacy of Craiova, 200349 Craiova, Romania; mihaela.mitroi48@gmail.com; 4 Department of Toxicology “Akademik Danilo Soldatović”-Faculty of Pharmacy, University of Belgrade, Vojvode Stepe 450, 11000 Belgrade, Serbia; aleksandra.buha@pharmacy.bg.ac.rs; 5 Department of Physiology, “Carol Davila” University of Medicine and Pharmacy, 050474 Bucharest, Romania; costin.caruntu@gmail.com (C.C.); cristian.scheau@umfcd.ro (C.S.); 6 Department of Dermatology, “Prof. N. Paulescu” National Institute of Diabetes, Nutrition and Metabolic Diseases, 011233 Bucharest, Romania; 7 Laboratory of Human Pathologies Biology, Department of Biology, Faculty of Sciences, Mohammed V University in Rabat, 10106 Rabat, Morocco; boyahyaa-90@hotmail.fr; 8 Genomic Center of Human Pathologies, Faculty of Medicine and Pharmacy, Mohammed V University in Rabat, 10106 Rabat, Morocco; 9 Laboratory of Histology, Embryology and Cytogenetic, Faculty of Medicine and Pharmacy, Mohammed V University in Rabat, 10100 Rabat, Morocco; nasrelomari@gmail.com; 10 Laboratory of Physiology, Pharmacology & Environmental Health, Faculty of Sciences, University Sidi Mohamed Ben Abdellah, 30000 Fez, Morocco; Nawal.ELMENYIY@usmba.ac.ma; 11 Toxicology Department, University of Medicine and Pharmacy of Craiova, 200349 Craiova, Romania

**Keywords:** cannabinoids, brain injury, neurodegenerative disorders, animal models, molecular mechanisms

## Abstract

Neurological disorders such as neurodegenerative diseases or traumatic brain injury are associated with cognitive, motor and behavioural changes that influence the quality of life of the patients. Although different therapeutic strategies have been developed and tried until now to decrease the neurological decline, no treatment has been found to cure these pathologies. In the last decades, the implication of the endocannabinoid system in the neurological function has been extensively studied, and the cannabinoids have been tried as a new promising potential treatment. In this study, we aimed to overview the recent available literature regarding in vivo potential of natural and synthetic cannabinoids with underlying mechanisms of action for protecting against cognitive decline and motor impairments. The results of studies on animal models showed that cannabinoids in traumatic brain injury increase neurobehavioral function, working memory performance, and decrease the neurological deficit and ameliorate motor deficit through down-regulation of pro-inflammatory markers, oedema formation and blood–brain barrier permeability, preventing neuronal cell loss and up-regulating the levels of adherence junction proteins. In neurodegenerative diseases, the cannabinoids showed beneficial effects in decreasing the motor disability and disease progression by a complex mechanism targeting more signalling pathways further than classical receptors of the endocannabinoid system. In light of these results, the use of cannabinoids could be beneficial in traumatic brain injuries and multiple sclerosis treatment, especially in those patients who display resistance to conventional treatment.

## 1. Introduction

Neurological disorders that affect different subsets of neurons such as neurodegenerative diseases (NDs) are associated with several cognitive, motor and behavioural changes that influence the quality of life of patients and the evolution and prognosis of the disease [1,2,3]. Similar changes have also been observed in those suffering from traumatic brain injury (TBI) or haemorrhagic/ischemic stroke [4,5]. Cognitive decline and motor impairments are usually associated with patients affected by neurodegeneration [6]. Cognitive decline is characterized by a decrease in executive function, attention and working memory [7], while in motor decline appear extrapyramidal rigidity, spasticity, motor impairment and gait problems [8]. The cognitive, behavioural and motor impairment is determined by the death of neurons in different regions of the central nervous system (CNS), and unfortunately, it cannot be treated. Even if a lot of progress has been made to understand the mechanism implicated in the pathogenesis of cognitive and motor impairment in neurodegenerative and brain injuries [9,10,11], no treatment has been found to cure these pathologies. Several strategies and therapies have been developed and tried until now to treat some symptoms and to decrease the neurological decline in order to improve the quality of life of patients [12], and many others underwent preclinical testing [13,14].

In the last years, the implication of the endocannabinoid (eCB) system in the neurological function has been extensively studied. The eCB system is formed from cannabinoid receptors, eCBs and enzymes implicated in the synthesis and metabolization of eCBs [15].

There are two cannabinoid receptors identified, cannabinoid receptor type 1 (CB1R) and type 2 (CB2R), that are coupled with G-protein and act through activation of mitogen-activated protein kinase (MAPK), modulating potassium channels and inhibiting voltage-gated calcium channels and adenylyl cyclases [15]. CB1R is localized mainly in the CNS in the cortex, caudate nucleus, globus pallidum, putamen, amygdala, hypothalamus, hippocamp, cerebellum, substantia nigra, and dorsal vagal complex, and CB2R is localized mainly in the periphery in immune cells and hematopoietic systems, such as spleen, leukocytes and tonsils, and mostly under pathological states in peripheral organs, such as testis, liver, muscle and intestine [16]. CB2R was also identified in CNS, mainly localized in microglia, astrocytes localized in the cortex [17], striatum [18], amygdala [19], hippocampus [20], cerebellum [21] and brainstem [22], but this expression is mostly observed in pathological conditions, possibly due to infiltration of peripheral cells or an increased expression on “activated” microglia or astroglia. CB2R is not usually identified in healthy brain cells. CB1R is mainly localized presynaptic, in the GABAergic terminals of peripheral and central neurons, being implicated in neurotransmitter release and psychoactivity by decreasing presynaptic GABA release that leads to a decrease in GABAergic inhibitory control of postsynaptic neurons, producing postsynaptic neurons exhibition [23]. In the periphery, CB1R is localized in testis, liver, uterus, adipose tissue and immune cells [24].

Studies showed that compared with CB1R, CB2R is mainly expressed in CNS after a specific insult such as anxiety, addiction and inflammation, while in resting microglia, no CB2R was detected [22]. CB2R are mainly localized postsynaptically in the CNS, and their activation produces hyperpolarization of membrane potential and inhibition of neuronal function having opposite effects compared with CB1R [25].

Cannabinoids are a group of chemicals that bind to the cannabinoid receptors acting as direct agonists, producing different effects according to the affinity to one or both cannabinoid receptors [26,27]. The cannabinoids are classified into three categories: i)endocannabinoids that are synthesized by mammals, ii)natural cannabinoids (phytocannabinoids) that are isolated from plants and iii) synthetic cannabinoids that are synthesized in the laboratory and have the same or all physiological properties of phytocannabinoids and endocannabinoids [28,29].

### 1.1. Endocannabinoids

2-arachidonoylglycerol (2-AG) and arachidonoyl ethanolamide (AEA, anandamide) are two of the best-studied endocannabinoids that bind to the CB receptors and produce effects similar to the psychoactive compounds found in cannabis [26]. AEA is a lipid neurotransmitter that has the ability to stimulate several receptors such as presynaptic and postsynaptic CB1R, CB2R, 5-hydroxytryptamine and Transient Receptor Potential Vanilloid 1 (TRPV1) ion channel. 2-AG is synthesized from glycerol and omega-6 fatty acid arachidonic acid and has a higher affinity for CB1R and CB2R when compared to AEA [30].

In recent years, other compounds that bind to the cannabinoid receptors have been identified such as 2-arachidonoyl glyceryl ether (noladin ether, 2-AGE), N-arachidonoyldopamine (NADA) and O-arachidonoylethanolamine (virodhamine) [26]. 2-AGE was the first compound isolated from porcine brain and considered as an ether linked-analogue of 2-AG. It is able to inhibit the intracellular accumulation of AEA and bound to CB1 and weakly to CB2 [31].

Virodhamine is formed from two molecules of arachidonic acid and ethanolamine, which are linked by an ester bond, and was detected in peripheral tissues and brain. It shows to be a partial agonist; more exactly, it acts in vivo as an antagonist at CB1R level and agonist of CB2R level [32]. NADA is an endocannabinoid that binds to and activates TRPV1 but also acts as endogenous TRPM8 antagonist [33].

The concentration of endocannabinoids is regulated by different mechanisms such as degradation that involves reuptake into the presynaptic cell, followed by rapid hydrolysis by fatty acid amide hydrolase (FAAH) of the amide or ester bonds or metabolization through monoacylglycerol lipase (MAGL) only for 2-AG [34].

### 1.2. Phytocannabinoids

*Cannabis sativa* is a plant that contains at least 104 cannabinoids identified until now, with different structure and different functional profile [35]. Two of these components form the majority and have been intensely studied in the last years: delta-9-tetrahydrocannabinol (Δ9-THC) and cannabidiol (CBD). Other compounds such as cannabinol (CBN), cannabigerolic acid (CBGA), cannabigerol (CBG), cannabidiolic acid (CBDA), cannabichromene (CBC), cannabielsoic acid A, cannabicyclol (CBL), Δ9-tetrahydrocannabinolic acid (Δ9-THCA) and Δ9-tetrahydrocannabivarin (Δ9-THCV) form the minority of phytocannabinoids found in *Cannabis sativa* plant [35].

Δ9-THC is the most psychoactive molecule found in the cannabis plant. It is a partial agonist of CB1R and CB2R and shows several physiological properties similar to endocannabinoids [26]. Its psychoactive activity is determined by the activation of CB1R in CNS, which leads to the inhibition of adenyl cyclase and decrease cAMP levels [30].

CBD is the non-psychotropic component isolated from cannabis, especially from fibre-type *Cannabis* species, and is used to treat several pathologies such as neurological diseases and cancer [26]. The affinity of this compound is low compared to Δ9-THC for the two receptors CB1R and CB2R and is considered as an inverse agonist of the human CB2R receptor [26]. The CBD exerts an inhibitory effect on the metabolism of tetrahydrocannabinol by blocking its conversion by cytochrome P-450 to give a more psychoactive molecule. Besides, it plays a counterbalancing role to regulate the negative effect of Δ9-THC produced by high doses consumption [36].

CBN was the first phytocannabinoid isolated from cannabis and is also the major metabolite of tetrahydrocannabinol. In general, this compound has a high affinity for CB2 receptors, but this affinity remains weak compared to that of tetrahydrocannabinol, and it is considered as a partial agonist of CB1R receptors [30].

CBDA is the non-psychoactive precursor of CBD, found in the fresh cannabis plant [30]. CBDA has the same capacity as CBD to antagonize TRPM8 receptor and to stimulate the transient receptor potential (TRP) cation channels, TRPV1 and TRPA1 with significantly less potency than CBD [37].

CBG is the major phytocannabinoid that has been found in several *Cannabis* varieties [38] acting as a CB2R antagonist and low CB1R antagonist similar to CBD [30]. It also acts as an agonist of α2-adrenergic and 5-HT1A receptors [30].

CBC is found in high amount in dry cannabis plants. In association with other cannabinoids such as CBD and Δ9-THC, it produces antidepressant effects and also promotes neurogenesis [39]. CBC is also an essential stimulator of transient receptor potential (TRP) ankyrin 1-type (TRPA1) channels [39].

Δ9-THCV is an n-propyl analogue of Δ9-THC that have similar pharmacological effects and molecular targets. This compound is a partial agonist of CB2R and GPR55 and also has the capacity to activate 5HT1A receptors and different TRP channels [38]. Other phytocannabinoids found in *Cannabis sativa* plant have been isolated and identified, but their activity and molecular targets have not been revealed so far.

### 1.3. Synthetic Cannabinoids

This class of compounds is the most diverse group concerning the chemical structure and biological activities. These are classified into seven structural groups: aminoalkylindoles (JWH-018, JWH-073 and AM-2201), tetrahydrocannabinols (Δ9-THC, HU 210), naphthylmethylindoles (JWH-185), cyclohexylphenols (CP47,497), naphthoylpyrroles (JWH-030), phenylacetylindoles (JWH-250, RCS-4), and naphthylmethylindenes (JWH-176)[40]. The synthetic cannabinoids have full agonist activity with a higher affinity to CB1R and CB2R receptors and higher dose-dependent efficacy compared with Δ9-THC [41].

In this study, we aimed to review the recent available literature regarding the in vivo potential of cannabinoids in protecting against cognitive decline and motor impairments that appear in acute brain injury and chronic brain injury. There are several chronic brain injuries produced especially by neurodegeneration, so in order to overview the changes that can appear in the modulation of cognitive decline and motor impairment between acute and chronic injuries, we chose as a model for chronic brain injury multiple sclerosis (MS). MS is defined as a chronic inflammatory demyelinating disease of the CNS associated with several degrees of degeneration and loss of axons [42]. Having also an autoimmune component, MS can affect both young and old people [42]. 

## 2. Methodology

Multiple searches on Pub Med were performed for the articles published between January 2009 and December 2019 using as keywords: “cannabinoids” or “cannabis”, or “brain injury” or “multiple sclerosis”. After analysing the results, only the relevant studies were included.

Inclusion criteria for the articles were in vivo studies on animal models of TBI or MS that analyse cognitive and motor impairment, written in English.

Exclusion criteria for the articles were articles written in languages other than English, studies published only as abstracts, reviews and meta-analysis, in vitro studies, human case reports, human clinical trials. For the review articles, their references were checked in order to identify significant ones.

## 3. Results

**Figure 1:**
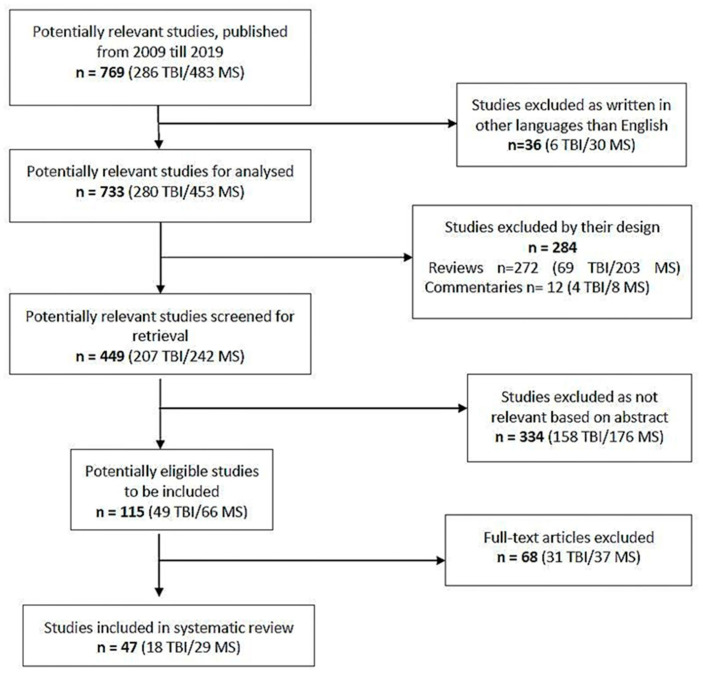
PRISMA flow chart of the study adapted after [43].

After an initial check, we identified 769 studies, 286 for TBI and 483 for MS. After we excluded the articles written in other languages than English, 733 articles remained to be analysed, 280 for TBI and 453 for MS. The reviews, commentaries and editorials were excluded, and 449 articles remained to be checked for relevance. After abstract analysis, 115 articles, 49 for TBI and 66 for MS were included and reviewed by reading the full text. Finally, 47 studies, 18 for TBI and 29 for MS were included in the analysis. The selection procedure is detailed in the PRISMA flow chart (Figure 1).

### 3.1. Cannabinoids Effects in Cognitive and Motor Impairment in Traumatic Brain Injury (TBI)

In Table 1 are presented in vivo studies published between 2009 and 2019 that evaluate the cannabinoids protective effects in TBI models (Table 1).

We identify 13 studies that evaluate the protective effects of synthetic cannabinoids active as CB2R agonists as HU-910 and HU-914 [44], JWH133 [45,46,54], GP1a [47], SMM-189 [1,49,51], ACEA [52], 0-1966 [57,60], KN38-7271 [59], ACEA [52] in TBI animal models, 2 studies that evaluated the protective effect of natural cannabinoid CBD [48,58] in TBI animal models, 1 study that evaluated the beneficial effect of exogenous administration of the AraS endocannabinoid in TBI animal models [56] and 2 studies that evaluated the protective effect of increasing the endocannabinoid levels in TBI animal models by inhibiting the enzymes that metabolize the endocannabinoids as FAAH [53] and ABHD6 [55].

In vivo animal studies showed that CB2R receptor is the main pharmacological target of natural or synthetic cannabinoids in protection against cognitive and motor impairment after TBI [1,44,45,46,47,48,49,51,54,55,57,58,59,60]. The inhibition of CB1R by SR141716 failed to show any beneficial effect on cognitive and motor impairment after TBI [50].

The main mechanisms through which cannabinoids improve cognitive and motor impairment after TBI are associated with decreasing the pro-inflammatory markers [1,44,46,47,48,52,53,57,58], decreasing oedema formation [44,45,47,54,55,58,60], decreasing BBB permeability [44,45,46,54,55,57], preventing neuronal cell loss [44,49,51,55] and increasing the levels of adherens jonction proteins [45,46,54].

### 3.2. Cannabinoids Effects in Cognitive and Motor Impairment in Multiple Sclerosis (MS)

In Table 2 are presented in vivo studies published between 2009 and 2019 that evaluate the cannabinoids protective effects in MS models (Table 2).

We identified 5 studies that evaluated the efficacy of the combination of Δ9-THC+CBD in decreasing clinical symptoms of EAE [61,62,63,64,65]; 6 studies that evaluated the efficacy of CB2R agonists in decreasing clinical symptoms of EAE [66,67,68,69,70,71]; 2 studies that evaluated the efficacy of exogenous administration of endocannabinoids or endocannabinoids-derivatives in MS [72,73]; 1 study that evaluated the efficacy of CB1R agonists in decreasing clinical symptoms of EAE [74]; 10 studies that evaluated the efficacy of CB1R, CB2R agonists in decreasing clinical symptoms of EAE [62,75,76,77,78,79,80,81,82,83] and 5 studies that evaluated the protective effect of increasing the endocannabinoid levels in EAE animal models by inhibiting the enzymes that metabolize the endocannabinoids as ABHD6 [84,85] and MAGL [86,87,88].

In vivo animal studies, the pharmacological target of natural and synthetic cannabinoids in decreasing clinical symptoms and progression of EAE are diverse and differ according to the murine model used.

Except for CB1R and CB2R receptors, the stimulation of PPAR-α and GPR55 receptors by cannabinoids is also associated with beneficial effects in EAE models [64,70,71,72,78,81,82,83].

The mechanisms through which cannabinoids decrease motor disability in MS murine models are diverse: decreasing the neuroinflammation [61,62,64,66,67,70,72,73,74,75,76,77,78,80,81,82,85], increasing anti-inflammatory cytokines [66], decreasing cell proliferation [66], increasing remyelination and axonal protection [68,86], decreasing demyelination [70,78,82,85], decreasing microglia activation [63,64,69,70,71,74,79,81,85,88].

## 4. Discussion

### 4.1. Cannabinoids Effects in Cognitive and Motor Impairment in Traumatic Brain Injury (TBI)

TBIs is often caused by traumatism and can lead to death. Various treatments such as the use of corticosteroids, barbiturates, hyperventilation, mannitol, hypothermia and cerebrospinal fluid drainage were developed for reducing the damage induced by brain injuries and improving the quality of life of these patients. However, until now, no specific therapy is available for patients who are refractory to conventional treatments, as the mechanisms involved in these pathologies are different and not yet deciphered [90].

Several critical investigations showed that the mentioned treatments exhibit different secondary damages, and therefore, their effects are not significant in the improvement of the treatment of brain injuries. In this way, several pieces of research indicated that novel approaches should be established based on the target, specifically damage generated by injuries [91]. The secondary damage is essentially due to the excitotoxicity induced high concentrations of glutamate and activation of its receptor. This produces an accumulation of intracellular calcium levels, which activate numerous destructive pathways such as reactive oxygen intermediates, caspases and calpains [92]. Another factor that seems to contribute to the management of brain injury is the endothelin. Indeed, the function of endothelin is well understood, which is to be involved in the cerebral circulation. By intermediate of the endothelin-A receptor, endothelin induces vasoconstriction to reduce blood flow and contributes therefore to the genesis of haemorrhagic stroke and ischemia [90].

Various in vivo studies reported that some synthetic, endogenous and natural cannabinoids exhibit effectiveness on several complications caused by brain injuries (Table 1).

The treatment with synthetic CB2R agonists immediately after injury showed promising results in recovery of the neurobehavioral deficits and sensorimotor impairment mainly due to the modulation of inflammatory markers [1,44,45,46,47,57], decrease of oedema formation [44,45,47,54,60], influence in the permeability of BBB [44,45,46,54,57], increase in adherence junction proteins [45,46,54] and prevention of neuronal loss [44,49,51].

Short term treatment with CBD in mice model of BCCAO has been shown to prevent motor and cognitive impairment through a complex mechanism related with stimulation of synthesis in the hippocampus of the levels of BDNF and MAP-2 proteins and stimulation of neurogenesis. All these are associated with a decrease in neuroinflammation and neuronal loss in the hippocampus [48,58].

2-AG and AEA are endocannabinoids found in high amount in the CNS and overexpressed after TBI. 2-AG is highly metabolized by MAGL and in less amount by ABHD6. AEA is metabolized by FAAH [93]. Studies showed that the inhibition of monoacylglycerol lipase determined a high increase of 2-AG brain level associated with CBR desensitization and tolerance. In contrast, ABHD6 inhibition produces an increase in 2-AG levels in a range associated with no side effects [94]. The use of WWL70, an inhibitor of ABHD6, showed protective effects in TBI by decreasing the lesion volume associated with 2-AG effects as CB1R agonist [55]. Regarding the neuroprotective effects of 2-AG, these have been associated with activation of both CB1R and CB2R and increasing the phosphorylation of ERK1/2 and AKT kinase implicated in cell survival [55]. The same effects were also observed for PF3845, an inhibitor of FAAH that is associated with an increase of AEA level in the brain [53].

**Figure 2:**
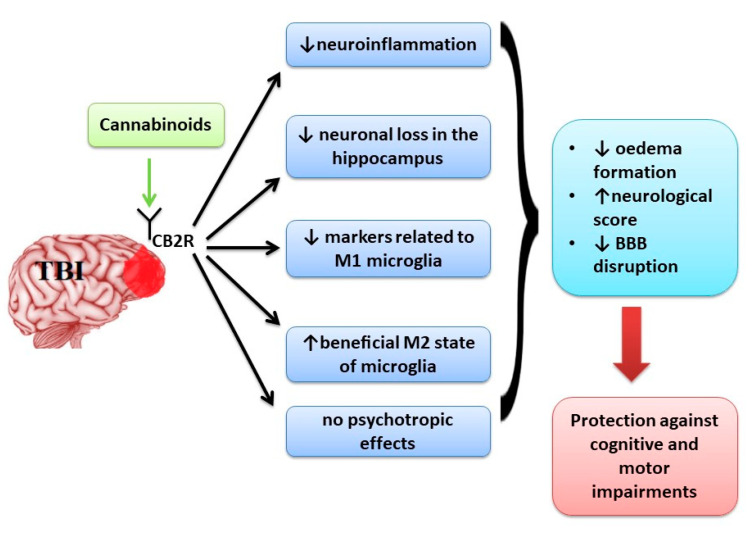
The main effects of activation of CB2R in TBI.

The activation of both CB receptors plays a protective role in cognitive and motor impairments observed in TBI by decreasing the oedema formation, improving neurological score and decreasing BBB disruption (Figure 2). Regarding the effect of CB receptors in microglia activation, CB2R activation has a protective role [95]. CB2R is associated with modulation of activated microglia [96]. There are two states of activated microglia, M1 state in which an increase production of reactive oxygen species (ROS) and inflammatory markers is observed and M2 state which is an anti-inflammatory state associated with healing processes [97]. Lopez-Rodriguez et al. [96] analysed the implication of CB receptors in minocycline protective effects on TBI and showed that the activation of CB1R is less associated with protective effects on TBI compared with CB2R. This effect is related to the fact that microglial cell activation leads to up-regulation of CB2R expression, while CB1R is kept at low levels.

The activation of CB2R was associated with a decrease of markers related to M1 microglia [1,57,60] and an increase in the beneficial M2 state of microglia [49,51] promoting neuroprotective effects without psychotropic effects determined by CB1R activation (Figure 2).

Targeting CB2R can be a promising therapeutic approach for TBI as the receptor is localized primarily in the microglia and less in neurons, and their expression is increased after a lesion that determines microglia activation.

### 4.2. Cannabinoids Effects in Cognitive and Motor Impairment in Multiple Sclerosis (MS)

Multiple sclerosis (MS) is a chronic autoimmune inflammatory disorder affecting the central nervous system (CNS) [98,99]. Multiple sclerosis is the most common inflammatory neurological disease in young adults [100]. The Global Burden of Diseases, Injuries, and Risk Factors Study (GBD) quantified the global burden of MS concluding that this disease is not common but is a potentially severe cause of neurological disability throughout adult life with substantially increased prevalence in many regions since 1990 [101]. Globally, it has been estimated that two million people suffer from this disease, with over 75% of them being women [102]. The exact aetiology of MS is still rather unknown, and research has suggested the involvement of various genetic and environmental factors, probably modulated by their complex interactions [103]. It is a widely held view that MS is a T cell-mediated autoimmune disorder. Namely, when autoreactive T-lymphocytes cross the blood–brain barrier (BBB) and enter CNS, local inflammation occurs, and subsequently, this results in demyelination, gliotic scarring and axonal damage [104,105]. Depending on the localization of the CNS lesions, MS can present various signs and symptoms such as pain, spasticity, muscle spasms, headache, numbness, fatigue and depression [98].

In vivo studies showed that a large variety of synthetic, endogenous and natural cannabinoids have beneficial effects in improving clinical outcome and evolution of MS by targeting the endocannabinoid system. Cannabinoid-based treatment that acts both on classical targets of the endocannabinoid system as CB1R/CB2R but also on PPAR-γ and GPR5 receptors that modulate neuroinflammation can be beneficial in MS on a case by case basis [64,70,71,72,78,82,83].

A recent in vivo study investigating the effects of Δ9-THC+CBD in EAE model provided further evidence that this combination suppresses neuroinflammation [61]. It was suggested that neuroinflammation could be mediated through regulation of miRNA-mediated signalling pathways based on the results obtained in brain-infiltrating cells [61]. Indeed, the involvement of miRNAs in the pathology of MS has been recently reviewed, and modulators of miRNA expression or function were suggested as disease-modifying therapeutics [106]. In a study utilizing EAE-induced mice, treatment with CBD ameliorated the severity of the clinical signs, suppressed microglial activation and T-cell recruitment in the spinal cord of mice, presumably not acting through known CB1 and CB2 receptors [81].

Moreover, neuroprotective effects against glutamate excitotoxicity, one of the major determinants of neurodegeneration in MS [107], were shown for CBD through CB receptor-independent mechanisms [108]. Other studies in rats with induced EAE disease performed to explore the anti-inflammatory properties of CBD revealed attenuation of EAE disease by evidenced by significantly reduced clinical scores of paralysis, decreased in IL-7 and IFN levels, and reduction in T cell infiltration in CNS were shown [62,77,89]. Interestingly, however, the possible role of immunosuppressive myeloid-derived suppressor cells (MDSCs) in observed CBD-induced amelioration was suggested, since CBD treatment led to a profound increase of these cells in mice. Anti-inflammatory effects of CBD were also shown in the viral model TMEV of MS. This is associated with adenosine A2A receptors participation in some of these effects [79].

BCP is a selective CB2R agonist of natural origin with anti-inflammatory and antioxidant effects that showed beneficial effects on decreasing neuroinflammation in MS models that lead to the decrease of disease progression [66,67]. The administration of the antidepressant imipramine (IMP) in a mouse model of MS improves clinical outcome and pathological score through modulation of inflammatory cytokine levels. In combination with BCP, IMP exerts synergistic effects [66].

PEA is an endogenous compound with cannabinoid-like effects, without psychoactive effects. It acts mainly as G-protein-coupled receptor 55 (GPR55) agonist and by activation of PPAR-α [72,78]. After treatment with PEALut, obtained from PEA and luteolin, the clinical symptoms and motor disabilities decrease in a murine model of EAE by drug modulation of neuroinflammation [72]. Another study that analysed the efficacy of CBD and PEA alone and in combination showed that both CBD and PEA have protective effects on a mice model of MS, but in combination, their effects are antagonistic, explained mainly by opposite effects on GPR55 receptor [78]. This study suggests the possible implication of the GPR55 receptor in the beneficial effects of cannabinoids in MS.

Inhibition of ABHD6 enzyme that leads to increase of 2-AG in the CNS can have promising results in the treatment of MS. KT182 compound, an inhibitor of ABHD6, succeeded in decreasing the motor disability but failed to induce neuro-anti-inflammatory effects [84]. On the other side, WWL70 compound, another inhibitor of ABHD6, proved more promising results by increasing 2-AG levels and improving clinical manifestation of MS, mainly by increasing 2-AG levels in microglia/macrophage and acting on CB2R, leading to decreased microglia activation and decreased production of inflammatory markers and adhesion molecules [85].

CBG is a phytocannabinoid that attracted the attention of researchers due to the lack of psychotropic effects. VCE-003 is a quinone derivative of CBG that has been shown to act mainly as CB2R agonist associated with regulation of microglial neurotoxicity and as PPARγ receptor agonist implicated in decreasing neuroinflammation [70,71]. In vivo studies showed that this compound has promising effects in decreasing progression and motor impairment in MS murine models [70,71].

The study conducted to investigate the efficacy of different extracts of *Cannabis sativa* revealed that Δ9-THC, CBD and cannabinoid free extracts are all active in CREAE-induced mice at different phases of the disease, likely by distinct mechanisms [65]. These findings are also supported by in vivo studies that investigated the efficacy of Sativex-like combination of phytocannabinoids and each component alone conducted in two models of MS, one on mice infected with Theiler’s murine encephalomyelitis virus (TMEV), inducing demyelinating disease model of MS [64], and one on mice with induced EAE model [63].

The therapeutic potential of a Sativex-like combination to slow MS progression was supported by the data [63,64]. The results obtained in the group treated with single substances showed that CBD acts similarly to Sativex when alleviating motor deterioration acting through PPARγ receptors while other component, Δ9-THC, acts primarily through CB1R and CB2R and produces somewhat weaker effects in the model with mice infected with TMEV [64]. It is interesting that CBD alone has no effect on the other model of MS with the mice-induced EAE, while the Δ9-THC-BDS acts similarly to Sativex when alleviating motor deterioration acting through CB1R and decreasing cell aggregates produced by microglial activation [63]. The differences in the efficacy of cannabinoids between the MS murine models of mice infected with TMEV and mice induced EAE can be explained by the differences observed in the CBR in these models. CB1R is less expressed in the brain of EAE rats [109], while CB2R is up-expressed in mice infected with TMEV [110].

Interestingly, some studies showed the up-expression of CB1R and CB2R in the glial cells of patients suffering from MS, showing the implication of CBR in the pathogenesis of MS disease [111]. Modulation of CB2R in MS is mainly associated with anti-inflammatory effects.

**Figure 3:**
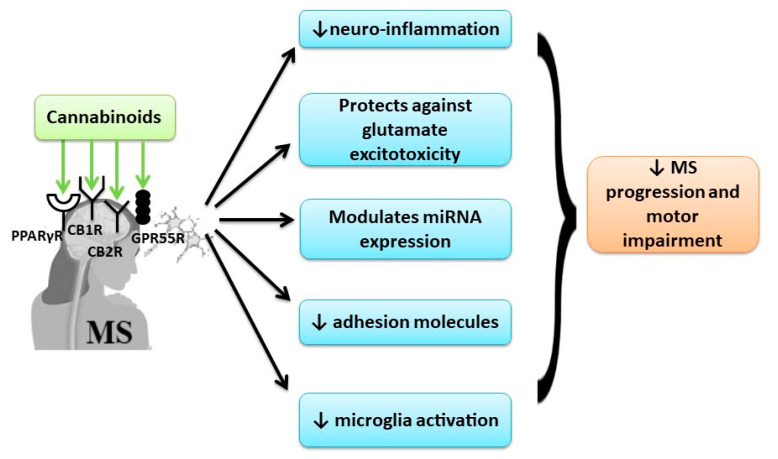
The beneficial effect of cannabinoids in MS by multi-receptor modulation.

The recent studies showed that using a broad-spectrum cannabinoid combination can have beneficial effects in MS treatment due to the implication of many targeting receptors, further than the classical receptors of the endocannabinoid system (Figure 3).

### 4.3. Limitations and Strength of the Study

The strength of this study is that it comprehensively summarizes the evidence from a large number of meta-analyses covering the impact of cannabinoids in protecting against cognitive decline and motor impairments that occur in acute brain injury and chronic brain injury.

A critical point of this review can be considered that the data were extracted and analysed from a large number of reviews and not from individual studies, although the meta-analyses provide the highest level of scientific evidence. No clinical trials were included, as the main purpose of this comprehensive review was to highlight the molecular mechanisms of action of cannabinoids in brain receptors and this is best done in animal models.

Even the research methodology can be considered a last limiting aspect; although it was systematic, this paper cannot be considered as a systematic review, because the quality of the studies themselves was not analysed in detail.

## 5. Conclusions

Based on benefits from cannabinoids use observed in animal studies, further clinical studies should be employed to proof the beneficial effect of cannabinoids’ treatment in TBI and MS, especially in those patients who are displaying resistance to conventional treatment.

The use of cannabinoids in TBI increases neurobehavioral function, working memory performance and decreases the neurological deficit and ameliorates motor deficit through down-regulation of pro-inflammatory markers, oedema formation and BBB permeability, preventing neuronal cell loss and up-regulating the levels of adherence junction proteins.

In murine models of MS, cannabinoids showed beneficial effects on improving clinical outcome, decreasing motor impairment and delaying the progression of the disease through down-regulation of neuroinflammation, up-regulation of anti-inflammatory cytokines, decreasing cell proliferation and decreasing demyelination and microglia activation.

In TBI treatment, targeting CB2R is a promising therapeutic approach, as the receptor is localized primarily in the microglia and less in neurons, and their expression is increased after a lesion that determines microglia activation. In contrast, in neurodegenerative diseases like MS, the cannabinoids showed beneficial effects in decreasing the motor disability and disease progression by a complex mechanism targeting more signalling pathways further that classical receptors of the endocannabinoid system.

## Figures and Tables

**Table 1:** Preclinical studies that show a correlation between cannabinoid treatment and cognitive and motor improvement in TBI animal models.

Type of Tested Cannabinoid	Doses	Receptors/Effects	Experimental In Vivo Animal Model	Cognitive Effects (Used Test)	Motor Effects (Used Test)	Mechanisms of Action/Results	Ref.
HU-910 HU-914 (camphor -resorcinol derivatives)	5–10 mg/kg (i.p.), 1 h after injury	CB2R agonists low CB1R affinity	C57Bl/6 WT mice CHI model	recovery of neurobehavioral deficits (NSS)	↑sensor motor recovery	↓inflammatory markers: ↓TNF-α, ↓IL-1α, ↓IL-1β, ↓IL-6 HU-914 showed the most important effects ↓TNF-α, ↓oedema ↓ BBB permeability ↓neuronal cell loss	[44]
JWH133	5.0 mg/kg (i.p.), 1 h after ICH	CB2R agonist	Sprague–Dawley rats CHI model	recovery of neurofunctional deficit (corner test)	↑spontaneous activity (sensorimotor Garcia test)	↓BBB breakdown ↓perihematoma ↓brain oedema ↑ adherens jonction proteins: ↑occludin, ↑ zo-1, ↑claudin-5	[45]
JWH133	1.5 mg/kg (i.p.), 1 h after surgery	CB2R agonist	Sprague–Dawley adult male rats CHI model	↑neurobehavioral outcomes (↓ NSS)	↑ motor impairment (forelimb placing test, corner turn test)	↓inflammatory markers: ↓IL-1β, ↓IL-6, ↓TNF-α, ↓ MMP2/9 ↑MKP-1→↓ MAPKs signalling pathway activation ↓neuroinflammation, ↑tight junction proteins: ↑zo-1, ↑cludin-5 ↓ BBB damage	[46]
GP1a	3 mg/kg bw 10 min before TBI	CB2R agonist	C57BL/6 mice cortical impact model of TBI	↓anxiety (OFT)	↑motor coordination (stationary beam walk, rotarod test)	↑anti-inflammatory markers ↑M2 macrophage polarization ↓cerebral oedema ↑ mean perfusion in the ipsilateral hemisphere	[47]
CBD	10 mg/kg (i.p.), 30 min before and 3, 24 and 48 h after surgery	CB1R, CB2R agonist	C57BL/6 mice model of BCCAO	↑cognitive performance (YM, OLT) ↓ anxiety (EZM) ↓ depression-like effects (FST)	↑ motor activity (OF)	↑nuclear receptors of the peroxisome proliferator-activated receptor family ↓adenosine uptake ↓reactive microglia and astrocytes, ↓ hippocampal neuroinflammation ↑BDNF protein, ↑DCX, ↑ MAP-2	[48]
SMM-189	6 mg/kg (i.p.), 2 h after model and then daily for 2 weeks	CB2R inverse agonist	C57BL/6J male mice mTBI	working memory in a spontaneous cross-maze alternation task,↓depression, ↓fearfulness ↑ cognitive and emotional deficits	mitigate functional deficits	↓ neuron loss preserve neuronal function and connectivity ↑beneficial M2 state of microglia	[49]
SR141716	10 mg/kg (i.p), 30 min after injury and then daily for 9 weeks	CB1R antagonist	Sprague–Dawley male rats TBI model	no improvement in spatial learning, memory (MWM)	no improvement (composite neuromotor score, beam-walking)	-	[50]
SMM-189	6 mg/, 2 h after injury and the daily for 14 days	CB2R inverse agonist	C57BL/6 male mice TBI model by single left-side blasts (50-60 psi)	-	↓motor deficits (Rotarod test)	↓ cortical and striatal neuron loss ↑ beneficial M2 state of microglia	[51]
ACEA	1 mg/kg, daily (i.p.) first within 5–1 min after modelling and then 1 per day for 6 days	CB1R agonist	Sprague–Dawley male rats TBI model	↑learning, ↑memory (NOR, MWT) No effects on anxiety (OFT, EPM)	no effects on locomotor coordination	↓neuroinflammation, modulate metabolic processes → preserved neuronal tissues or functions	[52]
PF3845 (a selective FAAH inhibitor)	5 mg/kg (i.p.) 30 min after TBI and then 1 per day for 14 days	CB1R, CB2R agonist	C57BL/6 male mice CCI model	↓ anxiogenic behaviour (EZM) ↑ working memory (YM)	↑ fine motor movements (BWBT)	↓ degenerating neuronal cells (dentate gyri) (CB1R and CB2R) ↑BcL-2, ↑Hsp72, ↑Hsp 70 ↓ COX-2, ↓ iNOS ↑Arg-1 in the ipsilateral cortex ↑ERK1/2, ↑AKT phosphorylation	[53]
SMM-189	6 mg/kg (i.p) 2 h after model and then 1/day for 14 days	CB2R inverse agonist	C57BL/6 mice mTBI model	↓depression (TST) ↓ fear response	↓motor deficit (Rotarod test)	↓ cytokines: IL-6, IFN –γ, IL-12p70, IL-10 ↓ Chemokines: IL-8, MIP-1β, TARC, MDC, eotaxin-3 ↓M1 microglial markers: CD11b, CD45, CD80	[1]
JWH133	1.0mg/kg 1 h after surgery	CB2R (agonist)	Male Sprague–Dawley rats SAH model	↓neurological deficits ↑neurological score ↑ neurobehavioral function (Garcia scoring system)	↓neurological deficits (Garcia scoring system)	↓brain oedema ↓BBB breakdown ↑ zo-1 ↓MPO ↑ TGF-beta1	[54]
WWL70 (selective inhibitor of ABHD6)	10 mg/kg (ip) 30 min after TBI and then 1 per day for 21 days	CB1R, CB2R agonist	C57BL/6 male mice CCI model	↑working memory performance (YM)	↑ fine motor movements (BWBT) ↑ motor coordination (Rotarod test)	↓lesion volume in the cortex (CB1R) ↓neurodegeneration in the dentate gyrus (CB1R and CB2R) ↓BBB breakdown ↓iNOS, ↓COX-2 ↑Arg-1 in the ipsilateral cortex ↑ERK1/2, ↑AKT phosphorylation	[55]
AraS	3 mg/kg (ip) 1 h after CHI model, after 7 days	CB1R, CB2R, TRPV1 channels	Sabra mice CHI model	↑neurobehavioral function (alertness, NSS)	↑neurobehavioral function (reflexes, coordination, motor abilities, balance) (NSS)	↓infarct volume ↓terminal differentiation of NPC into astrocytes ↑ neuroblast differentiation, ↑ doublecortin- neuroblastic marker	[56]
0-1966	5 mg/kg (ip) at 2, 24, 48 and 72 h after CCI	CB2R agonist	C57BL/6 mice CCI model	-	↑postinjury motor performance (rota-rod test) Open-field (forced exploration) testing	↓ BBB damage ↓ immune cell infiltration,↓ release of pro-inflammatory neurochemicals ↓ Iba-1-positive macrophages/microglia	[57]
CBD	1 mg/kg single dose	CB1R, CB2R agonist	Newborn Wistar rats HI model	↓ working memory impairment (NOR)	↑ motor coordination (Rotarod test); ↑motor deficit in the contralateral (right) forepaw (CRT)	↓ brain injury volume ↓ the extend of brain injury ↓ TNF-α ↓ oxidative stress	[58]
KN38-7271	0.1–10 mg/kg (i.p) 2 h before and 30 min, 4 h and on day 2 and 6 after MCAO	CB1R, CB2R agonist	Sprague–Dawley rat MCAO model		↓ MCAO-motor impairment (ladder rung walking test)	↓ cortical infarct size	[59]
0-1966	5 mg/kg (i.p) 1 h and 24 h after injury	CB2R agonist	C57BL/6 mice CCI model	↑exploratory behaviour (OFT)	↑Locomotor performance (rotarod, forelimb cylinder)	↓cerebral oedema ↓ perivascular areas of substance P immunoreactivity ↓ activated macrophages/ microglial cells	[60]

Abbreviations: ABHD6—alpha/beta hydrolase domain 6; ACEA—arachidonyl-2′—chloroethylamide; AraS—N-arachidonoyl-L-serine; Arg-1—Arginase-1; BBB—blood–brain barrier; BCCAO—by bilateral common carotid artery occlusion; BDNF—brain derived neurotrophic factor; BWBT—beam walk balance test; CBD—cannabidiol; CCI—controlled cortical impact; CHI—closed head injury model; COX-2—cyclooxygenase-2; CRT—cylinder rear test; DCX—doublecortin; EPM—elevated plus maze; EZM—elevated zero maze; FAAH—fatty acid amide hydrolase; FST—forced swim test; HI-hipoxia-ischemia; ICH—intracerebral hemorrhage; iNOS-inductible niitric oxide synthase; MAP-2—microtubule-associated protein 2; MCAO—middle cerebral artery occlusion; MDC—macrophage-derived chemokine; MIP-1β—macrophage inflammatory protein-1-beta; MMP2/9—matrix metallopeptidase-2/9; mTBI—mild traumatic brain injury; MWT—Morris water task; NOR—novel object recognition; NPC—neural progenitor cells; NSS—Neurological Severity Score; OFT—open field test; OLT—object location test; SAH—subarachnoid hemorrhage; TARC—thymus and activation-regulated chemokine; TRPV1—Transient Receptor Potential Vanilloid 1; TST—tail suspension test; YM—Y-maze; zo-1—zonula occludens-1.

**Table 2:** Preclinical studies that show a correlation between cannabinoid treatment and cognitive and motor improvement in MS animal models.

Type of Tested Cannabinoid	Doses	Receptors/Effects	Experimental In Vivo Animal Model	Motor Effects	Mechanisms of Action/Results	Ref.
Δ9-THC +CBD	10 mg/kg Δ9-THC and 10 mg/kg CBD in combination daily (i.p) from day ten until day 27	CB1R, CB2R agonists	C57BL/6 female mice induced EAE model	↓ clinical symptoms of EAE (several degrees of paralysis of hind limbs)	↓neuro-inflammation ↓miR-21a-5p, miR-31-5p, miR-122-5p, miR-146a-5p, miR-150-5p, miR-155-5p, miR-27b-5p ↑miR-706-5p, miR-7116	[61]
BCP alone or + IMP	5 mg/kg/day (p.o) BCP; 5/2.5 mg/kg/day (p.o) BCP+10 mg/kg/day IMP from day 10 to 37	CB2R selective agonist/sphingomyelinase inhibitor (IMP)	C57BL/6 mice induced EAE model	↓ clinical symptoms of EAE (more pronounce in the BCP+IMP groups)	↓pathological score (massive leukocyte infiltrations) (more pronounce in the BCP+IMP groups) ↓ inflammatory markers: TNF-α, IL-6, IL-17, IL-17/IL-10 ratio ↑ anti-inflammatory cytokines: IL-10 ↓ cell proliferation	[66]
PEALut (PEA + luteolin)	5 mg/kg/day from day 11 after modelling until day 27	PPAR-α and GPR55 receptor agonist	C57BL/6 mice induced EAE model	↓ clinical symptoms of EAE ↓ motor disability	↓TNF-α, IL-1β, IFN-γ, SAA1 mRNAs ↓ expression of receptors implicated in inflammation: CB2R, CD3-γ, Fpr2, TLR2, TCR-ζ chain	[72]
CanniMed oil Huile (10:10 and 1:20)	215 mg/kg oil extract (p.o) daily from day 6 to 18	CB1R, CB2R agonist	Lewis rats induced EAE model	↓ motor disability	↓TNF-α ↑BDNF protein expression	[62]
CBD	20 mg/kg (i.p) daily from day 9 to day 25	CB1R, CB2R agonist	C57BL/6 female mice induced EAE model	↓clinical scores of paralysis	↓ T cell infiltration in the CNS, ↓ IL-17, IFNγ	[62]
KT182 (pass BBB), KT203 (acts only in the periphery) (ABHD6 inhibitors)	2 mg/kg (i.p.) from day 1 of modelling until day 10	CB1R, CB2R agonist	C57BL/6 female mice induced EAE model	↓ motor disability (KT182) No effect on corticospinal tract conduction latency	No effects on inflammatory molecules KT182 prevent calcium overload to the mitochondria	[84]
CBD	10 mg/kg (i.p) daily from day 14 until day 28	CB1R, CB2R agonist	C57BL/6 male mice induced EAE model	↓clinical scores of paralysis	↑ phosphorylation of PI3K, ↑Akt, ↑mTOR, ↑ BNDF, ↑PPARγ ↓ pro-inflammatory cytokines: ↓IFN-γ, ↓ IL-17	[89]
UCM03025 (MAGL inhibitor)	5 mg/kg/day (i.p) from day 75 until day 85 after modelling	CB1R, CB2R agonist	SJL/J mice infected with TMEV	↓ motor disability	↓ astrogliosis ↓ CSPGs level in spinal cord ↑OPCs differentiation→remyelination and axonal protection ↓neuroinflammation	[86]
BCP	25/50 mg/kg twice/day (p.o) from day 0 until day 30	CB2R (selective agonist)	C57BL/6 male mice induced EAE model	↓clinical scores of paralysis ↓ progression of the disease	↓Iba-1 and iNOS ↓pro-inflammatory cytokines: TNF-α, IL-6, IL-1β ↓ CD4+, ↓ CD8+ T cells	[67]
2-amidoalkylindole derivatives	10 mg/kg/day or 30 mg/kg/day (i.p) from day 3 until day 19	CB2R selective agonist	C57BL/6 mice induced EAE model	↓ motor disability ↓clinical scores of paralysis (dose-dependent manner)	↓ leukocyte infiltration in the white matter region ↓ demyelination in the white matter	[68]
trans-(1-(1-(1H-1,2,4-Triazole-1-carbonyl)piperidin-4-yl)-4-benzo[d][1,3] dioxol-5-yl)-3-(4-fluorophenyl)azetidin-2-one (β-lactam-based MAGL inhibitor)	3 mg/kg/day (i.p) from day 6 after modelling until day 2	CB1R, CB2R agonist	C57BL/6 female mice induced EAE model	↓clinical scores of paralysis ↓ progression of the disease	-	[87]
PM226 (isoxazole derivative)	5 mg/kg/day (i.p) from day 1 until day 7 after modelling	CB2R selective agonist	SJL/J mice infected with TMEV	↓clinical scores of paralysis	↓ microglia activation (↓Iba-1+ cells)	[69]
CBD	10 mg/kg (i.p) daily from day 14 until day 28 after modelling	CB1R, CB2R agonist	C57BL/6 male mice induced EAE model	↓ motor disability	↓ activation of MAPK signalling pathway ↓claved-caspase 3 (marker of apoptosis) ↑ Bcl-2, ↓Bax ↓mitochondrial alterations	[76]
CBD	1% CBD-cream (CBD solubilized in propylene glycol and basis dense cream O/A daily for 28 days	CB1R, CB2R agonist	C57BL/6 mice induced EAE model	↓ motor disability (↓ paralysis of hind limbs)	↓ CD4 and CD8α T cells, ↓pro-inflammatory markers (Il-10, Foxp3, p-selectin, TGF-β, IFN-γ), ↓ cleaved caspase 3 (apoptosis) ↓iNOS, PARP, nitrotyrosine	[77]
CBD PEA	5 mg/kg/day CBD/PEA or CBD+ PEA (i.p) for 3 consecutive days from the first sign of disease	CB1R, CB2R; (CBD); PPARα and GPR55 agonist (PEA)	C57BL/6 female mice induced EAE model	↓ motor disability (CBD and PEA alone, no effect for CBD+PEA)	↓inflammation ↓demyelination ↓axonal damage ↓inflammatory markers: IL-17, TNF-α, IFN- γ	[78]
WWL70 (ABHD6 inhibitor)	10 mg/kg/day (i.p) from day 10 until day 21 or 28 after modelling	CB2R agonist (↑2-AG mainly in microglia/macrophage (activation of CB2R), not in T cell (activation of CB1R))	C57BL/6 female mice induced EAE model	↓ motor dissability ↓ disease progression	↓microglia/macrophage activation ↓inflammatory mediators: ↓(TNF-α, ↓iNOS, ↓COX-2 ↓demyelination ↓axonal damage	[85]
Sativex^®^ (Δ9-THC-BDS + CBD-BDS combination)/ Δ9-THC-BDS/ CBD-BDS	Sativex^®^ (1:1; 10 mg/kg/day from each)/20 mg/kg/day (Δ9-THC-BDS/ CBD-BDS) from day 11 until day 31 after modelling	CB1R (Sativex^®^ and Δ9-THC-BDS) agonist	C57BL/6 female mice induced EAE model	↓ motor disability ↓ disease progression (Sativex^®^ and Δ9-THC-BDS)	↓ cell aggregates determined by microglia activation (Sativex^®^ and Δ9-THC-BDS)	[63]
Sativex^®^ (Δ9-THC-BDS+ CBD-BDS combination)/ Δ9-THC-BDS/ CBD-BDS	Sativex^®^ (1:1; 5 mg/kg/day from each)/5 mg/kg/day (Δ9-THC-BDS/ CBD-BDS) (i.p) from day 70 until day 80 after modelling	PPARγ receptor agonist (CBD-BDS) CB1R (Δ9-THC-BDS)	SJL/J mice infected with TMEV	↓ motor disability ↓ disease progression (Sativex^®^ and CBD-BDS, less effects of Δ9-THC-BDS)	↓ infiltrates ↓ VCAM-1, ICAM-1 ↓ microglial activity ↓ pro-inflammatory cytokines ↓ axonal damage ↓ astrocyte reactivity and accumulation of CSPGs in the spinal cord	[64]
VCE-003	5 mg/kg/day from day 6 until day 27 after modelling	CB2R PPARγ receptor agonist	C57BL/6 female mice induced EAE model	↓ motor disability	↓ infiltrates ↓ CD4^+^ T cells in the spinal cord ↓ microglia/macrophage activation ↓ demyelination ↓ axonal damage ↓ inflammatory markers: ICAM-1, TNFα, iNOS, IFNγ, IL-17	[70]
2-(4-benzylphenyl)- acetate and 6-(biphenyl-4-yl)hexanoate derivatives (MAGL inhibitor)	5 mg/kg (i.p) from day 6 after modelling until day 27	CB1R, CB2R agonist	C57BL/6 mice induced EAE model	↓ motor disability	↑ 2-AG levels (spinal cord) →↓leukocyte infiltration and microglial responses, ↓ axonal damage, restore myelin morphology	[88]
CBD	5 mg/kg (i.p) daily from day 1 to 7 after infection	CB1R, CB2R, adenosine A2A receptors	SJL/J mice infected with TMEV	↓ motor disability	↓ VCAM-1, ↓chemokines: CCL2, CCL5 ↓ IL-1 ↓ microglia activation	[79]
CB52 (synthetic cannabinoid)	2 mg/kg/day (i.p) from day 3 until day 30 after modelling	CB1R agonist	C57BL/6 female mice induced EAE model	↓ motor disability	↓ microglia activation, ↓ nitrotyrosine formation, ↓T cell infiltration, ↓TNF-a ↓oligodendrocyte toxicity, ↓axonal damage, ↓myelin loss	[74]
WIN55,212-2	5 mg/kg/day (i.p) from day 11 until day 17	CB1R, CB2R agonist/ the effects are mediated more by CB1R	C57BL/6 mice induced EAE model	↓ motor disability ↓ progression of the disease	↓TNF-α, iNOS, COX-2 (spinal cord and brainstem)	[80]
VCE-003	5 mg/kg/day (i.p) for 14 days starting from day 60 after infection	PPARγ (partial agonist), CB2R (modest agonist)	SJL/J mice infected with TMEV	↓ motor disability	↓ microglial activation ↓ VCAM-1 ↓chemokines and chemokine receptors genes up-regulation	[71]
Δ9-THC -rich extract/ CBD – rich extract, Δ9-THC /CBD extract	50 mg/kg (i.p) Δ9-THC -rich Extract/CBD—rich extract; 25 mg/kg (i.p) Δ9-THC/CBD extract (acute treatment 1 dose on day 32 after modelling and chronic treatment for 7 days from day 68 after modelling)	CB1R, CB2R agonist	Biozzi AB/H mice CREAE model	↓ motor disability (chronic administration Δ9-THC-rich extract)	-	[65]
CBD	5 mg/kg/day (i.p) on days 19, 20 and 21 after modelling	not mediated via CB1R or CB2R	C57BL/6 female mice EAE model	↓ motor disability	↓ axonal loss ↓infiltration of T cells, ↓microglial activation	[81]
AEA	3.5 mg/kg/day (i.p) for 7 successive days from day 83 after modelling	CB1R, CB2R agonist	SJL/J mice infected with TMEV	↓ motor disability	↓ p35, p19 and p40 mRNAs ↓IL-17A	[73]
WIN55,212-2	10 mg/kg/day (i.p) for 15 days from the symptom’s debut	CB1R, CB2R agonist PPARγ receptor agonist	Dark Agouti female rat EAE model	↓ motor disability No relapse	↓ inflammatory load ↓ demyelination	[82]
WIN55,212-2	1.5 mg/kg (i.p) twice per day from day 1 for 3 successive days	CB1R, CB2R agonist PPARγ receptor agonist	SJL/J mice infected with TMEV	↓ motor disability	↓ adhesion molecules: ICAM-1, VCAM-1	[83]

**Abbreviations**: Δ9-THC—tetrahydrocannabinol; Δ9-THC-BDS—Δ9-tetrahydrocannabinol-botanical drug substance; AEA—anandamide; BCP—(−)-β-caryophyllene; CBD-BDS—cannabidiol-botanical drug substance; CD3-γ—T cell co-receptor CD3 γ chain; CREAE—chronic relapsing experimental autoimmune encephalomyelitis; CSPGs—chondroitin sulfate proteoglycans; EAE—autoimmune encephalomyelitis; Fpr2—N-formyl peptide receptor 2; GPR55—G-protein-coupled receptor 55; Iba-1—ionized calcium-binding adaptor molecule 1; ICAM-1—intercellular adhesion molecule 1; IMP—imipramine; iNOS- inducible nitric oxide synthase; MAPK—Mitogen-Activated Protein Kinase; MAGL—monoacylglycerol lipase; OPCs—oligodendrocyte progenitor cells; PEA—palmitoylethanolamide; PPARα—peroxisome proliferator-activated receptor alpha; PPARγ—peroxisome proliferator-activated receptor-gamma; SAA1—acute-phase protein serum amyloid A1; TCR-ζ chain—T cell surface glycoprotein ζ chain; TLR2—Toll-like receptor 2; TMEV—Theiler’s murine encephalomyelitis virus; VCAM-1—vascular cell adhesion molecule-1; VCE-003—cannabigerol quinone.
